# Supplementary figures and images for: A selection criterion for patterns in reaction–diffusion systems
Source: Theor Biol Med Model. 2014 Jan 29;11:7. doi: 10.1186/1742-4682-11-7 (PMC3925790; doi:10.1186/1742-4682-11-7)

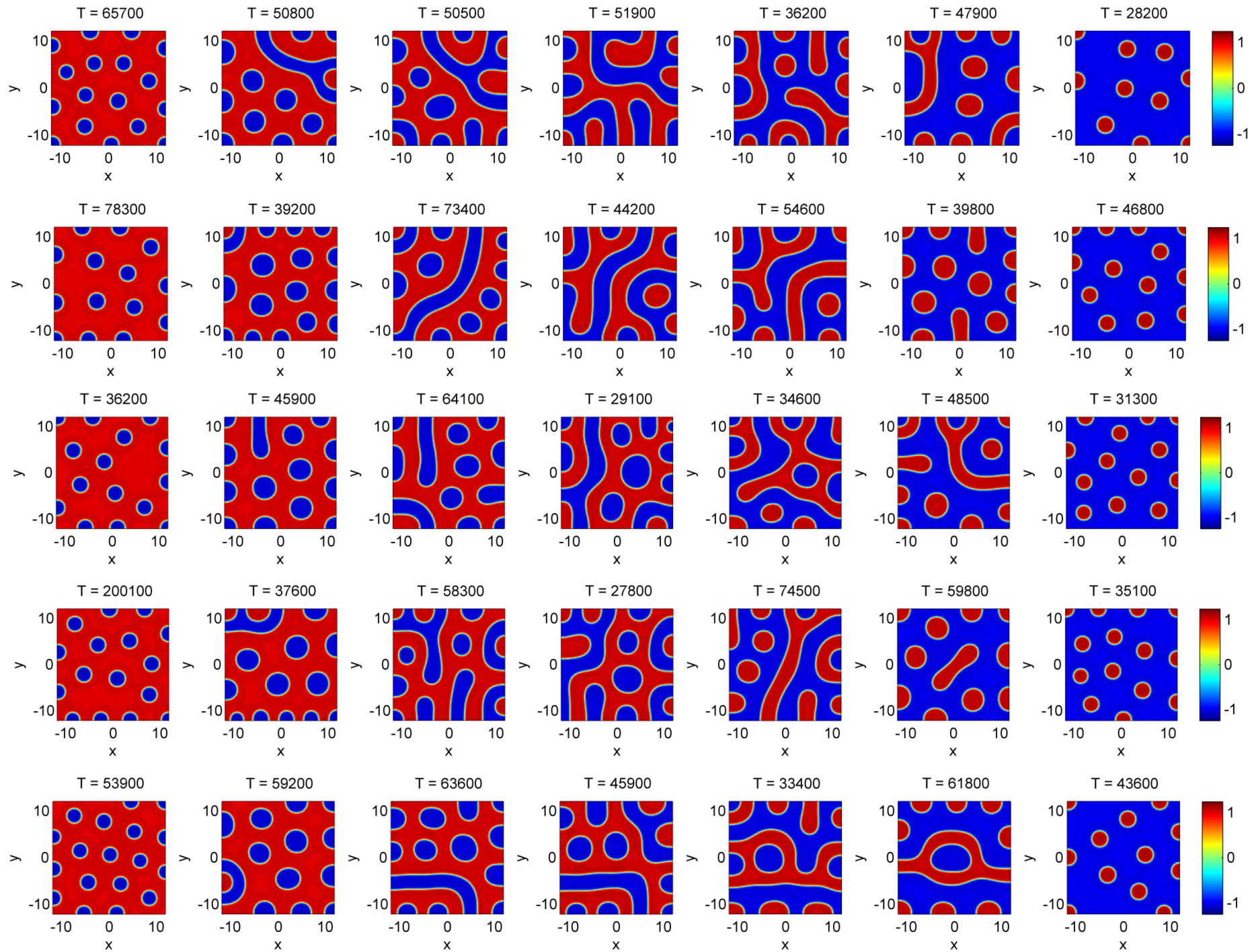

Supplement: Additional file 2: Figure S1 — Sample stationary solutions of Fitzhugh-Nagumo system with two morphogens in two spatial dimensions: pattern dependence on parameter R. Cases correspond to R = –0.04, –0.02, –0.01, 0, 0.01, 0.02, 0.04, from left to right. These cases correspond to the transition from inverted spots (R = −0.04, –0.02, –0.01) to labyrinths (R = 0) to spots (R = 0.01, 0.02, 0.04). All other simulation parameters D = 0.04, ϵ = 1, ρ = 0.3 are identical in all panels. Different rows correspond to uniform random initial conditions used, to ensure comparability between parameter variations. Simulations were run until a numerical steady state was reached (maximum difference in any lattice point less than 1e-16, the machine epsilon). Total time until reaching steady states are shown above each case. [file 1742-4682-11-7-S2.pdf]

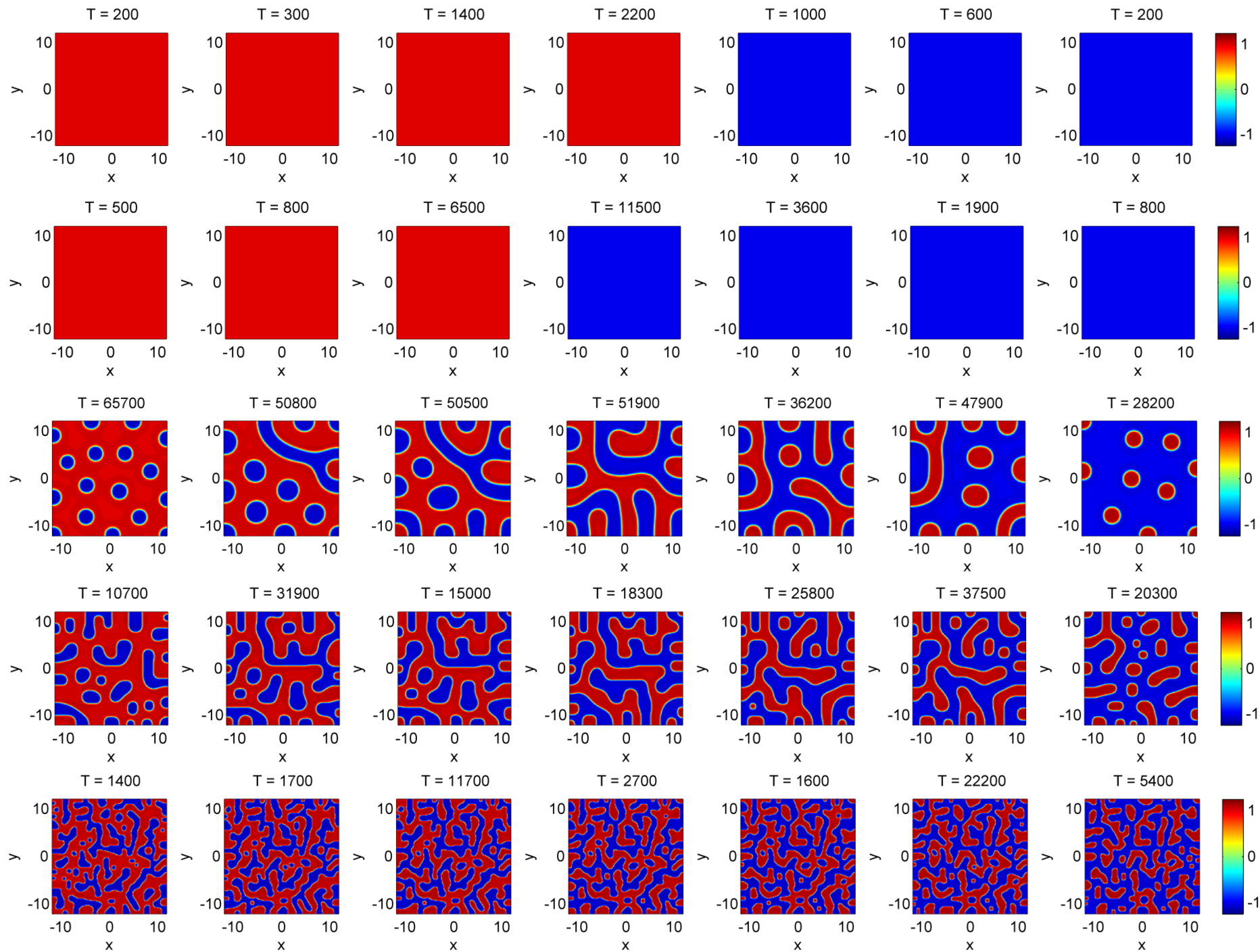

Supplement: Additional file 3: Figure S2 — Sample stationary solutions of Fitzhugh-Nagumo system with two morphogens in two spatial dimensions: pattern dependence on parameter D. Cases correspond to R = –0.04, –0.02, –0.01, 0, 0.01, 0.02, 0.04, from left to right. These cases correspond to the transition from inverted spots (R = −0.04, –0.02, –0.01) to labyrinths (R = 0) to spots (R = 0.01, 0.02, 0.04). Different rows correspond to distinct diffusion coefficients, D = 0.16, D = 0.08, D = 0.04, D = 0.02 and D = 0.01 from top to bottom. Identical random initial conditions were used in all cases, to ensure comparability between parameter variations. Simulations were run until a numerical steady state was reached (maximum difference in any lattice point less than 1e-16, the machine epsilon). Total time until reaching steady states are shown above each case. [file 1742-4682-11-7-S3.pdf]

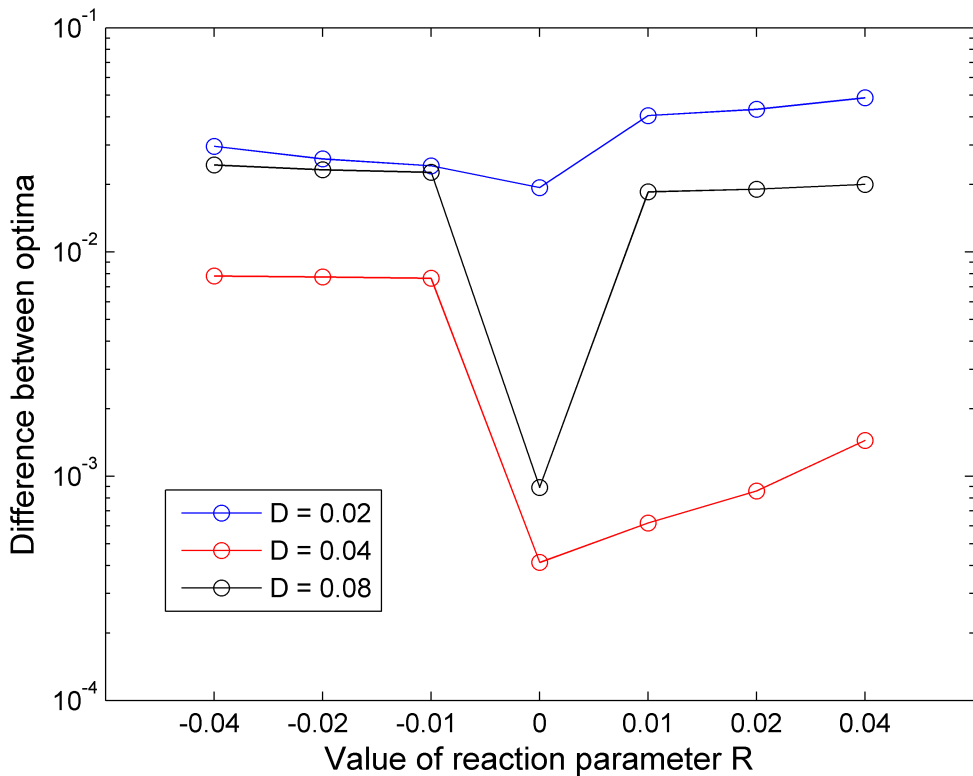

Supplement: Additional file 4: Figure S3 — Absolute differences between optima of Fokker-Planck equations associated to the Fitzhugh-Nagumo system. Color-coded curves correspond to distinct diffusion coefficients of morphogen u, while points within each curve correspond to distinct values of reaction parameter R In all cases, the Fokker-Planck equation was solved in Comsol, until steady state (total time T = 1000) with a time step of 0.01, and zero-flux boundary conditions. The initial condition was defined as ux,y,t=0=e−x2+y2 over the domain x, y ∈ [–5, 5]. [file 1742-4682-11-7-S4.pdf]
